# A Method to Simultaneously Detect the Current Sensor Fault and Estimate the State of Energy for Batteries in Electric Vehicles

**DOI:** 10.3390/s16081328

**Published:** 2016-08-19

**Authors:** Jun Xu, Jing Wang, Shiying Li, Binggang Cao

**Affiliations:** 1 School of Mechanical Engineering, Xi’an Jiaotong University, Xi’an 710049, China; jwang@mail.xjtu.edu.cn (J.W.); lishiying0927@stu.xjtu.edu.cn (S.L.); cbg@mail.xjtu.edu.cn (B.C.); 2 State Key Laboratory for Manufacturing Systems Engineering, Xi’an Jiaotong University, Xi’an 710049, China

**Keywords:** fault detection, state of energy estimation, battery model, proportional integral observer, battery management systems, electric vehicle

## Abstract

Recently, State of energy (SOE) has become one of the most fundamental parameters for battery management systems in electric vehicles. However, current information is critical in SOE estimation and current sensor is usually utilized to obtain the latest current information. However, if the current sensor fails, the SOE estimation may be confronted with large error. Therefore, this paper attempts to make the following contributions: Current sensor fault detection and SOE estimation method is realized simultaneously. Through using the proportional integral observer (PIO) based method, the current sensor fault could be accurately estimated. By taking advantage of the accurate estimated current sensor fault, the influence caused by the current sensor fault can be eliminated and compensated. As a result, the results of the SOE estimation will be influenced little by the fault. In addition, the simulation and experimental workbench is established to verify the proposed method. The results indicate that the current sensor fault can be estimated accurately. Simultaneously, the SOE can also be estimated accurately and the estimation error is influenced little by the fault. The maximum SOE estimation error is less than 2%, even though the large current error caused by the current sensor fault still exists.

## 1. Introduction

Due to pollution and the energy crisis, electric vehicles (EVs), including battery electric vehicles (BEVs), hybrid electric vehicles (HEVs) and plug-in hybrid electric vehicles (PHEVs) are of great importance [1,2,3]. In order to cope with the power and energy demands for such applications, a stable lithium-ion battery pack should be constructed with a large number of battery cells connected in series or parallel. A battery management system (BMS) is utilized to maintain optimum battery performance and ensure safety in EVs. As the key functions of the BMS, the State of Charge (SOC), the State of Health (SOH) and State of Energy (SOE) should be monitored online. As for the application of EVs, how much the remaining driving range of EVs left is a critical point that people should focus on. In fact, the SOE, which signifies the residual available energy in the battery, is more qualified than SOC to estimate the remaining driving range [4,5]. Unfortunately, SOE cannot be measured directly. As the most important information source for the SOE estimation, current sensor may fault, and the current information provided may have a bias. Consequently, accurate SOE estimation for batteries remains quite challenging and problematic.

An assortment of techniques has previously been reported to estimate the SOC of the batteries in EVs, each having its relative merits. The ampere-hour counting/integral (coulomb counting) method (ACM) is based on current measurement and integration, which is considered as the most common SOC estimation method. However, the prior knowledge of initial SOC is needed and it suffers from accumulated errors of noise and measurement error [6,7]. Although open-circuit voltage (OCV) method is very accurate, it needs a long rest time to estimate the SOC and thus it cannot be used in real time applications [6]. Besides, the sliding mode observer based estimations [8,9,10,11], the extended Kalman filtering (EKF) based estimators [12,13,14,15], the proportional integral observer (PIO) based estimations [16,17,18,19], the support vector based estimators [20], the neural networks (NNs) [21,22] and the fuzzy logic principle based estimations [23], etc. have been widely applied to estimate the SOC of batteries.

With respect to the SOE estimation, some studies have been conducted. The Back-Propagation Neural Network (BPNN) based method was utilized in [24] to estimate the SOE. The method considered the influence of the energy loss on the internal resistance, the electrochemical reactions, the decline of OCV, the discharge rate and the temperature fluctuation. However, the method is too complex to be widely implemented in practical applications. H. He et al. [25] chose the central difference Kalman filter (CDKF) and Gaussian model to estimate the SOE and concluded that the estimation error was small. Wang et al. [26] proposed a joint estimation approach based on PF algorithm to obtain the SOC and SOE respectively. However, the estimation accuracy of SOE relies on the prerequisite that the SOC has been well computed. Dong et al. [27] adopted dual filters-EKF plus PF-to estimate the SOE. The former filter is employed to update parameters of battery model on-line while the other filter is used to estimate the SOE. The recursive least square (RLS) with forgetting factor method was applied to identify the battery model and adaptive technique to estimate the SOE [28]. The simulation results demonstrated the effectiveness of the method. Barai et al. [4] presented a novel SOE estimation method based on the short-term cycling history.

Till now, the aforementioned methods have been studied and acceptable achievements have been made in different applications. However, most of the methods stated above were relied on the current information, namely the current sensor. What if the current sensor fails? Without proper actions, the estimated SOE will be useless, which may be dangerous for the batteries as over-charge or over-discharge may occur.

Actually, the application of fault detection and diagnosis is not new, extensive work in this area has been conducted with focus on different faults and related techniques. Permanent magnet demagnetization for IPMSMs was detected with nonsingular fast terminal-sliding-mode observer in [29]. Gang Huang et al. took advantage of sliding mode observer to detect the fault of the current sensor for PMSM drive systems [30]. In addition, fault detection is also becoming more and more important with efforts concentrated on detecting different battery faults. A. Izadian et al. proposed a special type of observer based fault detection technique, namely the multiple model adaptive estimation technique, and the performance shift in lithium ion batteries since over discharge could be detected [31,32]. Synthesized design of Luenberger observer (LO) was adopted in [33], along with equivalent circuit model (ECM) for fault isolation and estimation. The LO is a suitable candidate for fault detection in systems with little or no measurement noise. However, with the presence of noise, this setup has been confronted with inherent difficulties, particularly with performance variations. An adaptive model based technique was used to diagnose the over-charge and over-discharge faults in Li-ion batteries [34]. The authors adopted a conditional three-parameter capacity degradation model in [35]. Battery parameter identification, estimation and prognosis methodology were presented using several techniques, e.g., neural network (NN), auto regressive moving average (ARMA), fuzzy logic (FL) and impedance spectroscopy (IS), etc. [36].

With the support of these methods, the stated faults can be detected before the faults can go to extreme conditions. However, it is obvious that the related faults stated above are mostly about battery itself, but few is about sensor, especially about the current sensor used for SOE estimation. Satadru Dey et al. took advantage of three sliding mode observer to estimate three sensor faults independently [37]. Zhentong Liu et al. realized the detection and isolation of five faults, including the current sensor fault [38]. However, most studies are satisfied when the faults are detected. How to fix the fault or how to take measures to reduce the influence of the faults still remains to be solved.

In this work, the proportional integral observer (PIO) is introduced to simultaneously detect the current sensor fault and estimate the SOE. By taking advantage of the unique properties of the PIO, the current fault could be isolated from the state estimation, and thus, accurate current sensor fault could be obtained. Besides, with the accurate estimated results, the fault influence could be effectively eliminated. As a result, the SOE estimation could be accurate and robust, even the current sensor fault has occurred. The remainder of this paper is organized as follows: In Section 2, the simplified battery model is introduced and the state space expression of the battery model is deduced. The PIO based current sensor fault detection and SOE estimation method are proposed and analyzed in Section 3; Section 4 establishes simulations and experiments validation and the results are analyzed. Conclusions are provided in Section 5.

## 2. Battery Model Building and Analysis

**Figure 1:**
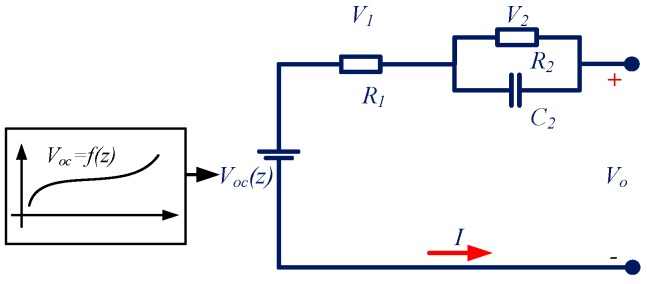
The simplified battery model.

In order to achieve a reliable battery state estimation and fault detection, an accurate model must be built first. Taking accuracy and computation complexity into consideration, the simplified battery model is introduced to characterize the battery. The schematic diagram for the simplified battery model is shown in Figure 1. As shown in the figure, several commonly used electric components are utilized. The OCV is adopted to describe the voltage source; series resistance ($R_{1}$) is used to describe the electrical resistance of various battery components; the diffusion resistance ($R_{2}$) and the diffusion capacitance ($C_{2}$) consisting of a RC network are adopted to describe the mass transport effects and dynamic voltage performances; the load current I is assumed positive for charge while negative for discharge; $V_{o}$ and $V_{1}$ is the terminal voltage and the voltage over $R_{1}$ respectively, $V_{2}$ describes the diffusion voltage over the RC network. The battery OCV is denoted as $V_{oc}(z)$ in this model to describe the OCV under different SOEs, where z is the abbreviation for battery SOE.

According to the circuit theory, Equations (1) and (2) could be obtained to describe the electrical relationship of the different parameters in the model.

(1) $${\dot{V}}_{2}=-\frac{1}{R_{2}C_{2}}V_{2}+\frac{1}{C_{2}}I$$

(2) $$V_{o}=V_{oc}(z)+V_{1}+V_{2}$$

According to the definition of SOE, which is the ratio of the remaining energy to the nominal energy, the mathematical relationship can be written as Equation (3).

(3) $$z(t)=z(0)+\Delta z=z(0)+\frac{1}{E_{n}}\int_{0}^{t}P(\tau )d\tau$$

where z(0) and z(t) are abbreviations of the initial SOE and the SOE at time t respectively; Δz is the variation of battery SOE during the time period from 0 to *t*, P(τ) is the instantaneous battery power, and $E_{n}$ is the nominal battery energy. Since z(0) is a constant for any given situation, Equation (3) can be rewritten as:

(4) $$\dot{z}=\frac{1}{E_{n}}P$$

If $V_{2}$ and z are chosen as the states of the battery model, the state function can be written as:

(5) $$\left\{ \begin{array}{l} {\dot{V}}_{2}=-\frac{1}{R_{2}C_{2}}V_{2}+\frac{1}{C_{2}}I\\ \dot{z}=\frac{1}{E_{n}}P=\frac{V_{o}}{E_{n}}I \end{array}\right.$$

For a given nonlinear system, the relationship between SOE and $V_{oc}(z)$ can be divided into several sections, and the subsystem in each section is considered to be linear [39]. Thus, the relationship can be written in the short SOE interval as follows for the *i*th SOE interval $(i-1)\cdot \Delta_{SOE}\leq SOE{}_{i}\leq i\cdot \Delta_{SOE}$:

(6) $$V_{oc}=a_{i}\cdot SOE{}_{i}+b_{i}$$

where $\Delta_{SOE}$ is the SOE interval length.

According to the explanation above, the output Equation can be described as:

(7) $$V_{o}=V_{oc}+V_{1}+V_{2}=a_{i}\cdot z+b_{i}+R_{1}\cdot I+V_{2}$$

The state space function with the additional state z can be rewritten as:

(8) $$\{\begin{array}{l} \dot{x}=Ax+Bu\\ y=Cx+Du \end{array}$$

where $A=\left[\begin{array}{cc} -\frac{1}{R_{2}C_{2}} & 0\\ 0 & 0 \end{array}\right]$, $B=\left[\begin{array}{c} \frac{1}{C_{2}}\\ \frac{V_{o}}{E_{n}} \end{array}\right]$, $C=\left[\begin{array}{cc} 1 & a_{i} \end{array}\right]$, $D=R_{1}$, $x=\left[\begin{array}{c} V_{2}\\ z \end{array}\right]$, $y=V_{o}-b_{i}$, u=I.

## 3. The Method to Simultaneously Detect the Current Sensor Fault and Estimate the SOE Based on Proportional Integral Observer

In this section, the current sensor fault is assumed to be a delta value between the actual current and the measured current. Such delta current fault is denoted as current sensor fault f. Given that the battery model is affected by the current sensor fault, the state space Equation is given as follows:

(9) $$\{\begin{array}{c} \dot{x}=Ax+B(u+f)\\ y=Cx+D(u+f) \end{array}$$

where $x\in {\mathbb{R}}^{2\times 1}$ represents the battery states, which is $V_{2}$ and z as stated in previous section, $y\in {\mathbb{R}}^{1\times 1}$ is the measured output, $u\in {\mathbb{R}}^{1\times 1}$ is the system’s input, which is the current I in the battery model, f represents the current sensor fault. A, B, C and D are known coefficients matrices with appropriate dimensions, which could be identified from the test data of batteries.

In order to take advantage of the unique properties of the PIO, the PIO is applied to the battery model and the observer is designed as follows:

(10) $$\left\{ \begin{array}{l} \dot{\tilde{x}}=A\tilde{x}+Bu+K_{p}(y-\tilde{y})+K_{i2}\tilde{f}\\ \dot{\tilde{f}}=K_{i1}(y-\tilde{y})\\ \tilde{y}=C\tilde{x}+Du \end{array}\right.$$

**Figure 2:**
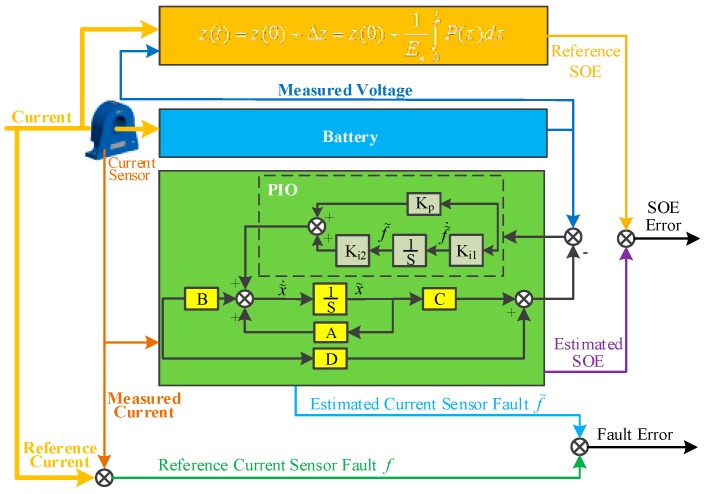
The method to simultaneously detect the current sensor fault and estimate the SOE.

Note that variable $\tilde{f}$ is defined as the integral of the difference $\left(y-\tilde{y}\right)$, which represents the estimated current sensor fault. Vectors $K_{p}\in {\mathbb{R}}^{2\times 1}$ is the proportional gain, $K_{i1}\in {\mathbb{R}}^{1\times 1}$ and $K_{i2}\in {\mathbb{R}}^{2\times 1}$ are the integral gains. The design block of the PIO based current sensor fault detection and compensation is shown in Figure 2.

It is supposed that the fault affecting the system is bounded. The expressions of the state reconstruction error and the fault reconstruction error are provided by the Equations as follows:

(11) $$\left\{ \begin{array}{l} e_{x}=x-\tilde{x}\\ e_{f}=f-\tilde{f} \end{array}\right.$$

The dynamics of the state reconstruction error is given by the computation of ${\dot{e}}_{x}$ which can be written as:

(12) $${\dot{e}}_{x}=\dot{x}-\dot{\tilde{x}}=(A-K_{p}C)e_{x}+Bf-K_{i2}\tilde{f}-K_{p}Df$$

To estimate the current sensor fault, it is designed that $K_{i2}=B$, thus, the Equation (12) could be rewritten as follows:

(13) $${\dot{e}}_{x}=\dot{x}-\dot{\tilde{x}}=(A-K_{p}C)e_{x}+Be_{f}-K_{p}Df$$

The fault error estimation is given as follows:

(14) $${\dot{e}}_{f}=\dot{f}-\dot{\tilde{f}}=\dot{f}-K_{i1}Ce_{x}-K_{i1}Df$$

The following matrices are introduced:

(15) $$\left\{ \begin{array}{l} \varphi =\left[\begin{array}{l} e_{x}\\ e_{f} \end{array}\right]\\ \varepsilon =\left[\begin{array}{l} f\\ \dot{f} \end{array}\right] \end{array}\right.$$

From the Equations (13)–(15), the following Equation can be obtained:

(16) $$\dot{\varphi }=A_{e}\varphi +B_{e}\varepsilon$$

with:

(17) $$A_{e}=\left[\begin{array}{cc} A-K_{p}C & B\\ -K_{i1}C & 0 \end{array}\right]$$

(18) $$B_{e}=\left[\begin{array}{cc} -K_{p}D & 0\\ -K_{i1}D & I \end{array}\right]$$

where the matrix I is the identity matrix with appropriate dimensions. The convergence of error Equation can be proved by choosing the Lyapunov candidate function as follows:

(19) $$V=\varphi^{T}P\varphi$$

where *P* indicates a defined positive matrix. The state reconstruction error $e_{x}$ and the fault reconstruction error $e_{f}$ tend towards zero if $\dot{V}<0$ according to the Lyapunov theory.

(20) $$\dot{V}={\dot{\varphi }}^{T}P\varphi +\varphi^{T}P\dot{\varphi }=(\varphi^{T}A_{e}^{T}+\varepsilon^{T}B_{e}^{T})P\varphi +\varphi^{T}P(A_{e}\varphi +B_{e}\varepsilon )\;=\varphi^{T}A_{e}^{T}P\varphi +\varepsilon^{T}B_{e}^{T}P\varphi +\varphi^{T}PA_{e}\varphi +\varphi^{T}PB_{e}\varepsilon$$

It could be rewritten as:

(21) $$\dot{V}=\psi^{T}\Pi \psi$$

where $\psi =\left[\begin{array}{l} \varphi \\ \varepsilon \end{array}\right]$, $\Pi =\left[\begin{array}{cc} A_{e}^{T}P+PA_{e} & PB_{e}\\ B_{e}^{T}P & 0 \end{array}\right]$.

The resolution of the inequality Π<0 leads to the values of $K_{p}$ and $K_{i1}$. By these procedures, the differential of the Lyapunov candidate function is assured to be negative, which means when t→∞, estimated state $\tilde{x}$ would converge to true state x and the estimated fault $\tilde{f}$ will converge to the true fault f.

## 4. Simulation and Experimental Validation

**Figure 3:**
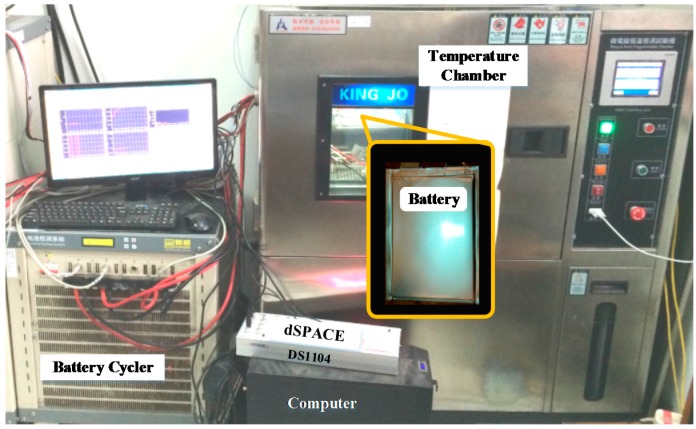
Experimental battery test work bench.

To verify the effectiveness of the proposed strategy for current sensor fault detection and compensation, the simulation and experimental validation are established. The experiment data used for this study are acquired through the battery test bench set up as shown in Figure 3, which consists of a battery cycler, a temperature chamber for environment control, a computer with dSPACE for performing the proposed method and experimental data storage.

**Figure 4:**
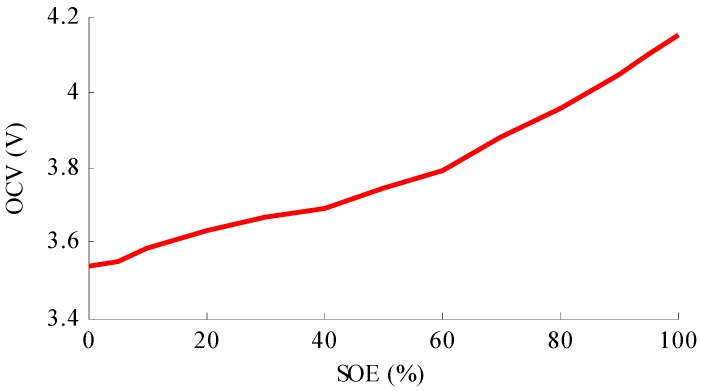
The relationship between OCV and SOE.

As shown in the figure, the battery cycler, which is controlled by the computer with TCP/IP, is used to apply current profile to the battery. Meanwhile, the voltage, current and temperature are measured by the battery cycler and the data will be sent to the computer. It is assumed in the paper that the message measured by the battery cycler is relatively true, and the stored data will act as the reference signals. For example, the current will be applied to calculate the SOE and the reference SOE could be obtained. The battery is placed in a temperature chamber to simulate the temperature environment of the battery when in practical implementation. The dSPACE DS1104 control board is utilized in this paper to perform the proposed algorithm. The current signal is measured by the current sensor through an ADC, and the voltage of the battery is measured directly through another ADC of the control board. The proposed algorithm is conducted with the Matlab/Simulink environment. After the algorithm is validated with the simulation in the Matlab/Simulink environment, the model will be modified and downloaded to the dSPACE control board. The dSPACE control board performs the algorithm and sends the calculated data back to the computer, such as the estimated SOE, estimated current sensor fault etc. The nominal capacity of the tested battery is 20 Ah, with 3.7 V nominal voltage. The upper cut-off voltage and the lower cut-off voltage are 4.2 V and 2.75 V, respectively. Based on the tested data, the least square method is introduced to calculate the parameters of the battery model $R_{1}$, $R_{2}$ and $C_{2}$. The calculated parameters for the battery model are: *R*_1_ = 0.0027 Ω, R_2_ = 0.0042 Ω, C_2_ = 25000 F, $a_{i}$ = [0.0032, 0.0071, 0.0052, 0.0051, 0.0029, 0.0033, 0.0051, 0.0085, 0.0079, 0.0088, 0.0100, 0.0111, 0.0111] and $b_{i}$ = [3.5264, 3.5071, 3.5252, 3.5286, 3.5952, 3.5772, 3.4892, 3.281, 3.3244, 3.25, 3.1447, 3.044, 3.044], respectively. The relationship between OCV and SOE is shown as Figure 4.

The urban dynamometer driving schedule (UDDS) is an automobile industry standard vehicle time velocity profile for urban driving that has been used for a number of years for electric vehicle performance testing. The current profile computed from the UDDS and scaled to percent of peak discharge power is utilized to emulate the real power requirement for the battery when it is equipped in an EV. In this study, the peak discharge current was set to 4.5 C rate (90 A). The reference SOE profile is calculated using definition method with the measured data.

### 4.1. Simulation Study for the Proposed Method

In order to demonstrate the effectiveness of the proposed method, based on the above experimental results, the parameters of the battery model are calculated. The battery model is used to calculate the voltage response of the battery. Meanwhile, the same battery model parameters are also adopted in the proposed current sensor detection and SOE estimation method. The parameters for the method are: *K_p_* = [0.5; 0.5], *K_i1_* = [85], *K_i2_* = [8e − 5; 1.389e − 3], respectively.

**Figure 5:**
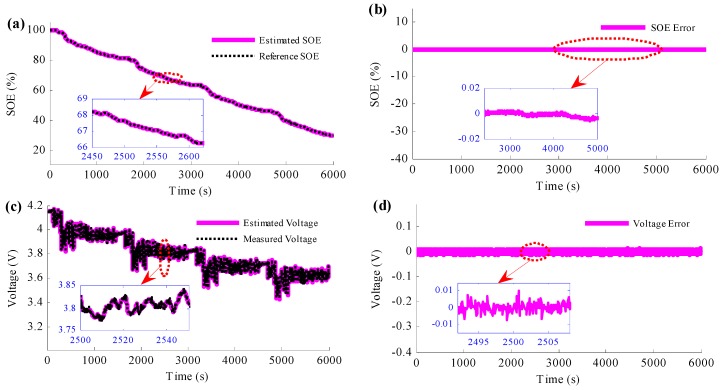
Simulation results when the current sensor works properly: (**a**) SOE estimation results; (**b**) SOE estimation error; (**c**) voltage results; (**d**) voltage estimation error.

Firstly, to verify the proposed method, the normal situation when the current sensor works well is firstly studied, as shown in Figure 5. In this situation, the battery is charged to full, which means that the initial SOE of the battery is 100%. Figure 5a,b are the simulation results of the estimated SOE of the proposed method, and the comparison between the estimated voltage and the measured voltage is shown in Figure 5c. The SOE estimation results show that the estimated SOE converges to the reference SOE, which has been proved in the previous section for the properties of the PIO. In addition, the SOE estimation error results also give the same conclusion. Besides, with the zoom figure of the SOE estimation error results, it is obvious that the estimated SOE traces the reference trajectory accurately, with less than 0.01% SOE estimation error. The main reason is that the battery model is very accurate in this simulation. The voltage estimation results show that the voltage prediction error is less than 10 mV, which is very accurate.

**Figure 6:**
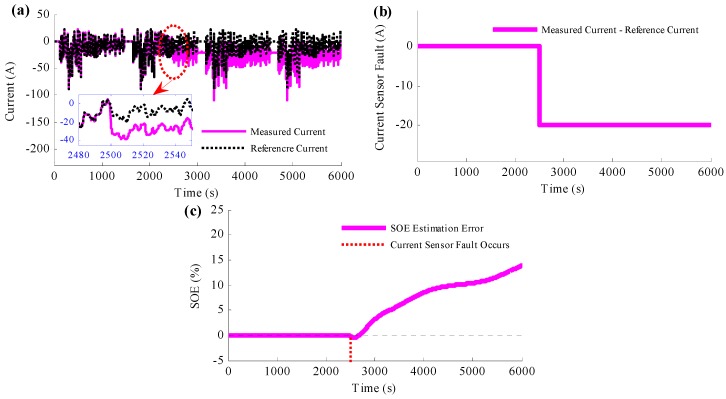
Simulation results when current sensor fails without taking any action: (**a**) measured current and reference current results caused by the current sensor fault; (**b**) current fault resulting from measured current minus reference current; (**c**) SOE estimation results when current sensor fault occurs and no action is taken.

To determine the influence of the current sensor fault to the SOE estimation, a special situation is set that the current sensor would fail at 2500 s. At this time, there will be a constant current error caused by the current sensor, which is set to be −20 A in this section, as shown in Figure 6a,b,c shows the SOE estimation results when current sensor fault occurs and no action is taken. It is obvious from the figure that the estimated SOE traces the reference SOE accurately before the current sensor fault occurs. At time 2500 s, when the current sensor fails without taking any action, the estimated SOE diverges from the reference SOE suddenly and the SOE estimation error becomes bigger and bigger. Since then, the estimated SOE has become not so accurate, and it cannot provide as the design and control reference any more.

**Figure 7:**
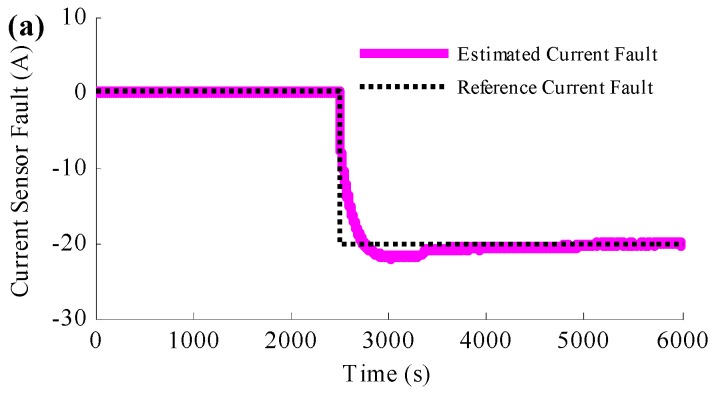

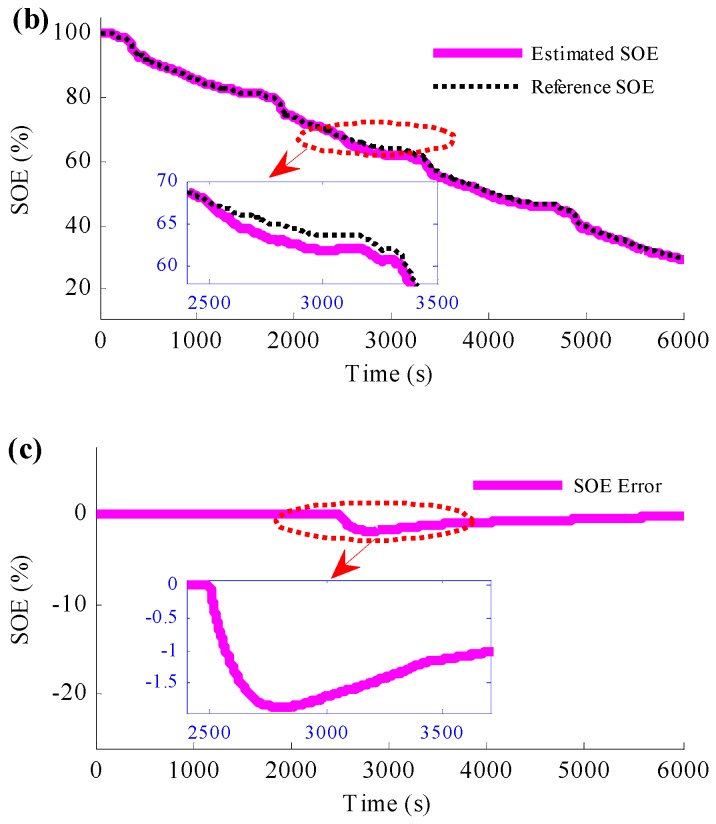
Simulation results when the method to simultaneously detect the current sensor fault and estimate the SOE is applied: (**a**) comparison between the estimated current fault and the reference current fault; (**b**) the SOE estimation results; (**c**) SOE estimation error.

To reduce the influence caused by the current sensor fault, the proposed simultaneous current sensor fault detection and SOE estimation method is applied to the simulation and the results are shown in Figure 7. Figure 7a is the comparative profiles between the estimated current fault and the reference current fault. Figure 7b presents the estimated SOE with the reference SOE when the current sensor fails and Figure 7c is the SOE estimation error. According to Figure 7a, it is found that the estimated current error converges to the reference current error after a short time; after the convergence, the estimated current error traces the reference trajectory accurately. It is clear from Figure 7b,c, when the current sensor fault occurs, the estimated SOE deviates from the reference SOE suddenly. However, thanks to the PIO based current sensor fault detection method, the estimated SOE converges to the reference SOE in a short time. In addition, since then, even the current sensor is still with large error, the estimated SOE could be stick to the reference SOE, which means the proposed method is not only able to estimate the current sensor fault, but also to compensate the fault. With such measures, the SOE estimation is robust to the current sensor fault.

**Figure 8:**
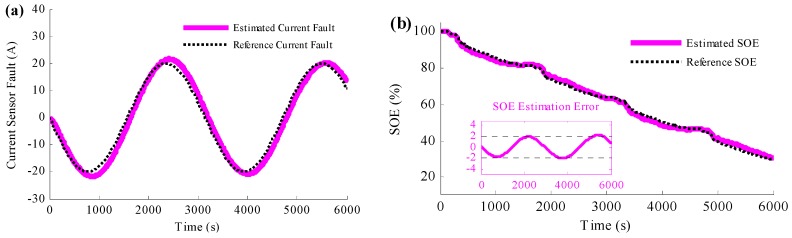
Simulation results when the current sensor fault is sinusoidal and the method to simultaneously detect the current sensor fault and estimate the SOE is applied: (**a**) the estimated current fault results; (**b**) the SOE estimation results.

To study more severe situation of the current sensor fault, the current error caused by the fault is assumed to be sinusoidal in the next simulation, and the results are shown in Figure 8. Figure 8a is the comparison between the estimated current fault and the reference current fault, and the estimated SOE is shown in Figure 8b. Based on Figure 8a, it could be concluded that the current sensor fault causes large current error, which owns 40 A peak-to-peak value. However, according to the figure, the estimated current error traces to the reference error accurately, even the reference current error changes sinusoidal. Figure 8b shows that, with such large current error, the proposed method could ensure the accuracy of the estimated SOE. The SOE estimation error is less than 2% even there are 40 A peak-to-peak value sinusoidal current faults.

**Figure 9:**
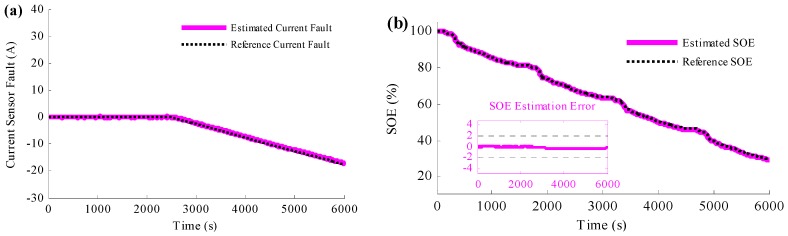
Simulation results when the current sensor fault is ramp-type and the method to simultaneously detect the current sensor fault and estimate the SOE is applied: (**a**) the estimated current fault results; (**b**) the SOE estimation results.

The ramp-type current error caused by the current sensor fault is also studied in the simulation, and the results are shown in Figure 9. The results depicted by the figures indicate that such ramp type current error could also be estimated and the SOE estimation could also be accurate.

From the simulation validation results, it can be concluded that the proposed method to simultaneously detect the current sensor fault and estimate the SOE is able to estimate accurate current error when current sensor fault occurs; besides, the method is also able to compensate the current sensor fault, and the influence on the SOE estimation caused by the fault can be eliminated in a short time, even severe situation is taken into consideration. The experimental validation with battery in loop will be discussed in the following section.

### 4.2. Experimental Validation for the Proposed Method

**Figure 10:**
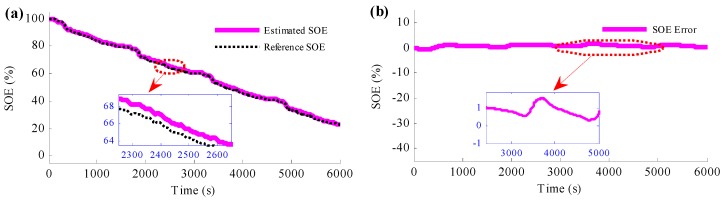
Experimental results when the current sensor works properly: (**a**) SOE estimation results; (**b**) SOE estimation error.

In this section, instead of battery model, an actual battery is utilized to experimentally validate the proposed method. The experimental validation workbench is established as shown in Figure 3. A current sensor is utilized to obtain the current information used for the state estimation method, while the current information measured by the battery cycler is assumed to be true current information in this study. Similar to the simulation, the normal situation when the current sensor works properly is firstly analyzed, and the results are shown in Figure 10.

Figure 10a shows the SOE estimation results when the current sensor is working properly, while the SOE estimation error is shown in Figure 10b. It is clear from Figure 10 that the method could get accurate SOE even the actual battery is tested in the experiment. The estimated SOE traces the reference SOE with small error, and the SOE estimation error is combined in 2% error bound.

To emulate the current sensor fault, a voltage bias is added to the current sensor output pins at 2500 s. By this action, since 2500 s, the current used for the SOE estimation method owns a certain error, which is −20 A in this study.

**Figure 11:**
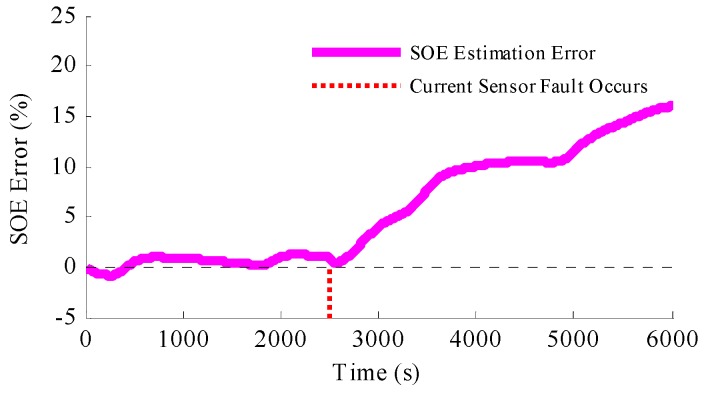
Experimental results when current sensor faults without taking any action.

If no action is taken when the current sensor fault occurs, the SOE estimation has large error as shown in Figure 11. It is obvious that when the current sensor fault occurs at 2500 s, the estimated SOE starts to deviate from the reference SOE immediately, and since then, the SOE estimation error becomes bigger and bigger.

**Figure 12:**
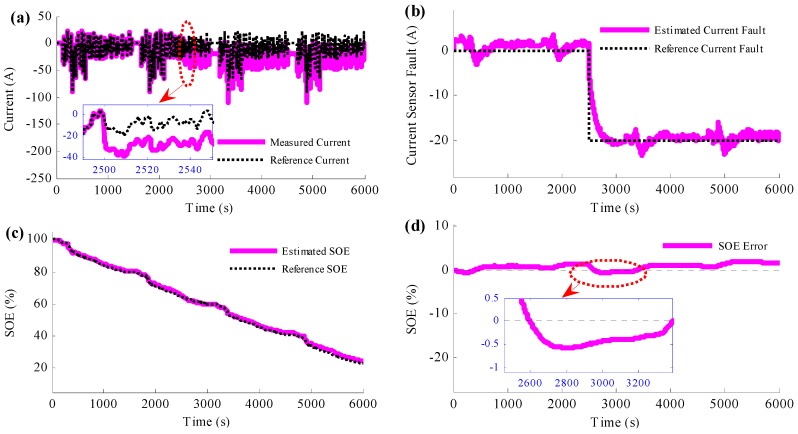
Experimental results when the method to simultaneously detect the current sensor fault and estimate the SOE is applied: (**a**) the current results; (**b**) comparison between the estimated current fault and the reference current fault; (**c**) the SOE estimation results; (**d**) the SOE estimation error results.

Figure 12 shows the results when the proposed current sensor fault detection and SOE estimation method is applied to the experiment. The true current and the measured current with current sensor fault are shown in Figure 12a. Figure 12b is the comparative profiles between the estimated current fault and the reference current fault. Figure 12c shows the SOE estimation results with the proposed method. Figure 12a and its zoom figure present that the current sensor fault provides the SOE estimation method with wrong current information. However, Figure 12b shows that the current error caused by the current sensor fault can be estimated accurately. The estimated current error traces the reference current fault well, even when there is a step change at 2500 s from 0 A to −20 A. The estimated current fault converges to the reference current fault in a short time, which provides the proposed method accurate information to compensate it. However, compared with the simulation results in Figure 7, the estimated error ripple is bigger when the actual battery is applied, which is believed to be caused by the battery modeling error.

As shown in the figure, the current sensor fault detection and SOE estimation method is realized simultaneously, and the estimation is accurate. Similar to the simulation results, the estimated SOE traces to the reference SOE accurately as shown in Figure 10c. When the current sensor fault occurs, the estimated SOE deviates from the reference SOE a little, and in a short time, the estimated SOE converges to the reference SOE. The maximum SOE estimation error is less than 2%, which exists exactly the time when the current sensor fault occurs. Henceforth, even the current sensor is still with large error, the estimated SOE sticks to the reference SOE.

## 5. Conclusions

A method to simultaneously detect the current sensor fault and estimate the SOE for batteries in EVs has been proposed in this paper. The simplified battery model was firstly introduced and analyzed. Secondly, the PIO based current sensor fault detection and SOE estimation method was proposed. The convergence of fault detection and state estimation has been proved. In order to give the efficiency of the proposed method without modeling error influence, the simulation of the proposed method was established first. The current error caused by the current sensor fault could be estimated accurately, and the SOE of the batteries could be obtained effectively. To further verify the proposed method, a battery test workbench was established and the proposed method was applied to an actual battery used in EVs. Through implementing the experiments, acceptable accuracy of the fault estimation and the SOE estimation has been verified. The current error caused by the current sensor fault could be accurately estimated, even when the error was severe. The influence caused by the current sensor fault could be eliminated in a short time and the biggest SOE estimation error was as small as 2%.
